# Electrochemical and theoretical studies of the interaction between anticancer drug ponatinib and dsDNA

**DOI:** 10.1038/s41598-024-52609-z

**Published:** 2024-01-27

**Authors:** Sylwia Smarzewska, Anna Ignaczak, Kamila Koszelska

**Affiliations:** 1 Department of Inorganic and Analytical Chemistry, University of Lodz, 12 Tamka Str, 91-403 Lodz, Poland; 2 Department of Physical Chemistry, University of Lodz, 163/165 Pomorska Str, 90-236 Lodz, Poland

**Keywords:** Electrochemistry, Medicinal chemistry, Theoretical chemistry

## Abstract

In this study, electrochemical and theoretical studies were performed to explain the interaction mechanism between ponatinib (**PNT**), a third generation tyrosine kinase inhibitor, and dsDNA. The electrochemical part was conducted in phosphate-buffered saline (PBS) at physiological pH of 7.4 and in acetate buffer with a pH of 4.7, using square wave voltammetry. A boron-doped diamond electrode was used in a bulk-incubated solution. The theoretical part was investigated using computational methods, such as the semiempirical method PM7 and density functional theory (DFT). Significant differences in the electrochemical behavior of **PNT** in the presence of DNA confirmed the occurrence of interactions. The results obtained in the acetate buffer strongly suggested the preferential interaction of **PNT** with guanine residues. However, at physiological pH, it can be concluded that **PNT** interacts with dGua and dAdo in the dsDNA molecule. These results are consistent with outcomes from the theoretical studies, where quantum-chemical calculations showed that both electrochemically detectable nucleobases form hydrogen bonds with the drug. These bonds appeared to be stronger with guanine than with adenine. According to the computational studies, the dsDNA major groove is the energetically preferred site for the complexation of **PNT**.

## Introduction

**Figure 1:**
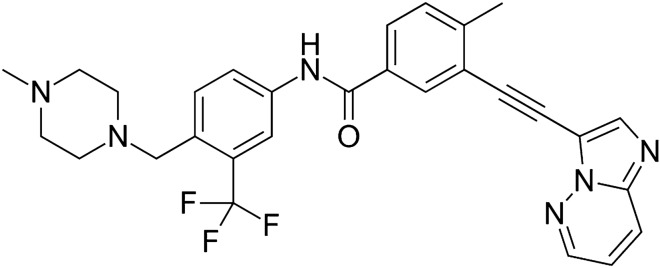
Chemical structure of **PNT.**

Tyrosine kinases (TK) are enzymes that act as ‘on’ and ‘off’ switches in many cellular processes, such as cell cycle regulation, proliferation, and cell death. They catalyze the transfer of the γ-phosphate of ATP to the tyrosine hydroxyl groups on target proteins^1^. Recent advances have implicated the role of TK in the pathophysiology of cancer. Although their activity is strictly controlled in normal, healthy cells, mutations or overexpression can lead them to acquire transforming functions that result in malignancy^2^. Constitutive oncogenic activation in cancer cells can be effectively blocked by selective tyrosine kinase inhibitors (TKI)^2^. Over the years, more than 20 TKIs effective against various cancers have been developed. Ponatinib (**PNT**, Fig. 1) is a multi-targeted third-generation tyrosine kinase inhibitor, approved in 2012 for the treatment of chronic myeloid leukemia (CML—a malignancy characterized by an elevated number of white blood cells^3^) and Philadelphia chromosome-positive acute lymphoblastic leukemia (Ph + ALL). In contrast to first and second generation drugs, such as imatinib, dasatinib, or nilotinib, **PNT** is also active against BCR-ABL1 T315l and other mutations^4,5^. Nonetheless, considering the serious cardiovascular risks, heart failure, and hepatotoxicity associated with its use, **PNT** is generally limited to cases of second-generation drug-resistant leukemia and/or as third-line therapy in CML^4–6^. Recently, it was shown that **PNT** treatment resulted in a marked and dose-dependent increase in double-strand DNA break damage^7^.

Based on its effectiveness against chronic myeloid leukemia, ponatinib is undergoing intensive evaluation for its potential activity against other tumor types, such as liver cancer and malignant pleural mesothelioma^7,8^. From a chemical perspective several methods have been developed to date for the analytical determination of **PNT** and its main metabolite in plasma or urine^1,9–13^. Generally, chromatography has been the most commonly used technique for the determination of **PNT** and other TKIs. However, considering the negative ecological impact of chromatography, electrochemical techniques are becoming increasingly popular for such analyses. To our knowledge, only one study has so far demonstrated the voltammetric approach to **PNT** determination^13^. The use of electrochemical techniques is of great importance, not only in analytical studies but also in increasing our understanding of the mechanism of drug action and its pharmacokinetics. The most commonly used technique in redox mechanism evaluation is cyclic voltammetry (CV). The power of cyclic voltammetry lies in its ability to provide substantial information on the kinetics of heterogeneous electron transfer reactions and coupled chemical reactions, as well as the thermodynamics of redox processes^14,15^. Square wave voltammetry (SWV) is one of the most sensitive methods for the direct evaluation of chemical compound concentrations. It can be widely used for the trace analysis of biologically active compounds. An important advantage of SWV is the possibility to detect, in a single scan, the reversibility of the electron transfer. In the past decade, there has been great interest in the electrochemical approach toward drug-DNA interaction studies^16–18^. Such studies, and the proper interpretation of the results, have contributed to the elucidation of the mechanism by which DNA may be damaged by hazardous compounds.

Bearing in mind that ponatinib may cause DNA damage, and the mechanism of this process is still not clear, the goal of this work was the extensive examination of the interactions between **PNT** and double-stranded DNA (dsDNA). The interaction studies were performed using voltammetric techniques, mainly SWV. In general, electrochemical studies were based on the analysis of differences in the electrochemical behavior of **PNT** in the presence and absence of dsDNA. The investigation and clarification of the electrochemical behavior of **PNT** were also performed using CV and SWV. In addition, the structural properties of the **PNT** molecule and its complexes with dsDNA in aqueous solution, as well as the strength of interaction between them, were investigated using computational methods, such as the semiempirical method PM7 and density functional theory (DFT). The information obtained from these studies will surely contribute to a better understanding of the mechanism of interaction of **PNT** with nucleic acids.

## Material and methods

### Chemicals and reagents

Low molecular weight double-stranded DNA (dsDNA) was purchased from Sigma (Germany). A stock solution of dsDNA was prepared daily by dissolving the required mass of DNA powder in phosphate-buffered saline (PBS; Sigma, Germany)) with a pH of 7.4. The prepared solution was stored at 4 °C and was used no longer than 24 h after preparation. Ponatinib was purchased from LGC Standards (UK). A stock solution of **PNT** was prepared by dissolving an appropriate mass of the compound in ethanol and was then stored at 4 °C. Solutions with lower **PNT** concentrations were prepared by proper dilution of the stock solution. Various supporting electrolytes, namely acetate, phosphate, Britton–Robinson (BR) buffers, and phosphate buffered saline, were also prepared and used for the studies. All buffer components were purchased from Avantor (Poland) and were of analytical grade. Deionized and distilled water was used for the preparation of the solutions.

### Apparatus and instrumentation

Electrochemical experiments were carried out using a μAutolab type III (Metrohm-EcoChemie, The Netherlands) coupled with an M164 electrode stand (mtm‐anko, Poland), operated with the GPES software (version no. 4.9). A conventional three-electrode system was used, comprising a boron-doped diamond working electrode (BDDE; Windsor Scientific Ltd, United Kingdom, diameter: 3 mm), a platinum wire as an auxiliary electrode, and an Ag/AgCl as a reference electrode. All studies were performed using a 0.01 L voltammetric cell.

The pH measurements of prepared buffer solutions were performed using a digital pH/mV/ion meter (Elmetron, Poland) with a combined glass electrode (Hydromet, Poland). Water was demineralized using a Polwater DL3 system (Labopol-Polwater, Poland).

### Electrochemical measurements

Before each voltammetric measurement, the BDD electrode surface was polished with alumina slurry on a polishing cloth. After the polishing procedure, the electrode surface was carefully rinsed with distilled and deionized water. All measurements were performed in triplicate, at the ambient temperature of the laboratory (21–23 °C).

Cyclic voltammetry and square-wave voltammetry techniques were applied for the general characterization of the electrochemical behavior of **PNT**. However, interaction studies were performed using only square-wave voltammetry. The SWV conditions used were as follows: amplitude of 30 mV, frequency of 25 Hz, and step potential of 4 mV.

Interaction studies: measurements of **PNT** (at its fixed concentration) were conducted in the presence and absence of DNA in acetate buffer at pH 4.7 and PBS at pH 7.4. After recording the voltammogram for the blank solution, a proper amount of **PNT** and dsDNA were added to a voltammetric cell. In the incubation procedure, **PNT** was mixed with dsDNA and then incubated at room temperature for different periods. After the predetermined incubation time, voltammograms were recorded. As a control experiment, both dsDNA and **PNT** were also analyzed separately, without mixing each other, in the used supporting electrolyte.

### Computational methods

The theoretical research included finding the most stable conformer of the **PNT** molecule, as well as examining the stability of its complexes with dsDNA. In both cases, a three-stage “hierarchical” approach was used, in which the accuracy of the theoretical method was increased at each subsequent stage. In the first step, the calculations were performed using the Amber99 force field of molecular mechanics. In the second step, the semiempirical method PM7 was used, and in the third step—the density functional theory (DFT) method M062X-GD3^19,20^ with the basis set 6-31G(d,p) and the Polarizable Continuum Model (PCM)^21^ of water was applied.

The procedure used in the conformational search conducted for the **PNT** molecule alone is shown in Fig. S1 and described in Electronic Supporting Information (ESI) (see the section Procedure S1 and Fig. S2 in the ESI). The final structures were obtained from the DFT optimizations in water (PCM). Among them, two different models of **PNT** were chosen to test their binding to dsDNA: the lowest energy, compact structure (denoted below as **PNT_LE**) and the entirely different, stretched geometry (**PNT_ST**).

To create the initial models of the dsDNA:**PNT** complexes, two B-DNA dodecamers (double helix) with different sequences were taken from the RCSB Protein Data Bank: 1BNA (5′-D(*CP*GP*CP*GP*AP*AP*TP*TP*CP*GP*CP*G)-3 3′))^22^ and 119D ((5′-D(*CP*GP*TP*AP*GP*AP*TP*CP*TP*AP*CP*G)-3 3′))^23^. Using the HyperChem program^24^, both structures were terminated with hydrogen atoms. To mimic the presence of counterions in a real environment, one hydrogen atom was added to each phosphate group. Thus, the total charge of each molecule was equal to 0. The two dsDNA models were then fully optimized in the program MOPAC^25^ with the PM7 method and the MOZYME procedure. In these calculations, the effect of the solvent (water) was included by using the Conductor-like Screening Model (COSMO)^26^. For these structures, single point energy calculations were performed in water using the DFT method M062X-GD3/6-31G(d,p)/PCM.

**Figure 2:**
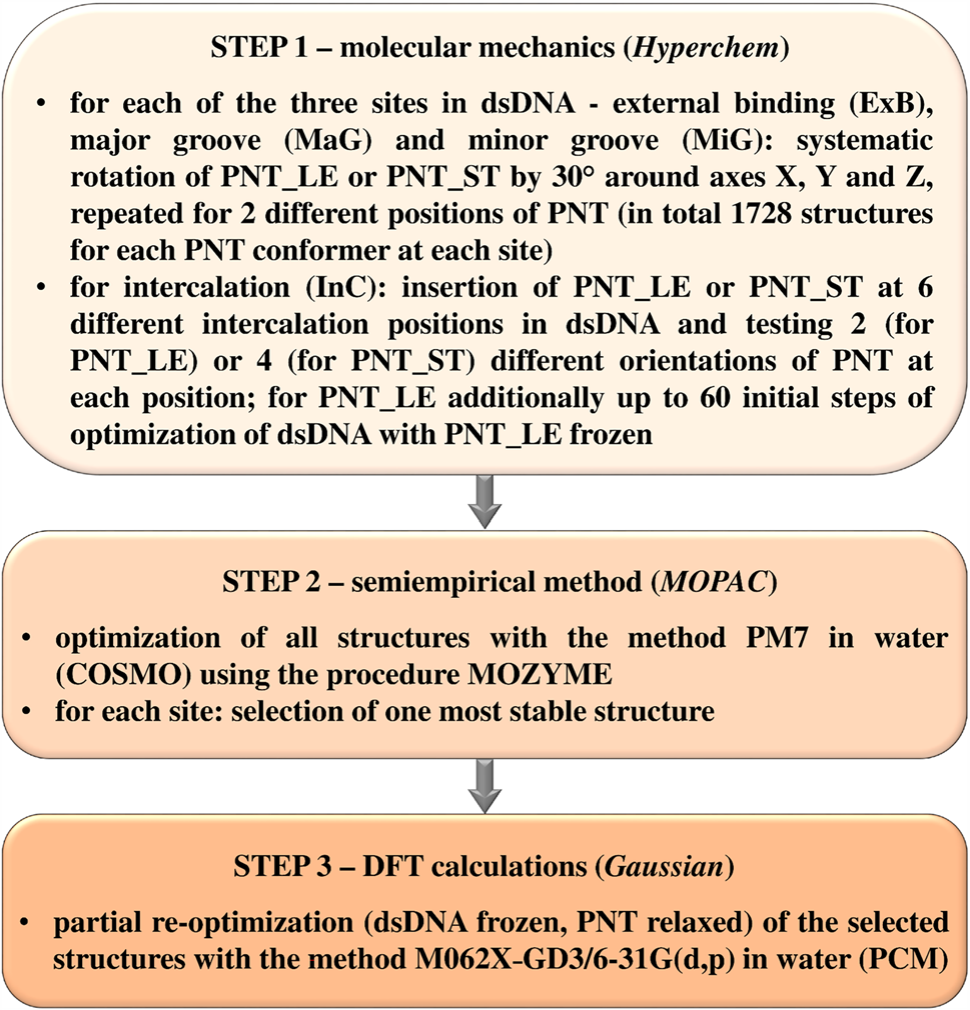
The flow chart of the procedure used to explore the stability of the dsDNA:**PNT** complexes.

To study the dsDNA:**PNT** complexes, for each of the two dsDNA models, four typical sites for binding **PNT** were considered: external binding (ExB), intercalation (InC), major groove (MaG) and minor groove (MiG). The final structures were obtained from partial optimizations (during which the dsDNA structure was kept frozen, while the **PNT** molecule was fully relaxed) performed with the M062X-GD3/6-31G(d,p) method in water (PCM) for the complexes selected after the semiempirical PM7 calculations. The calculations were performed using the Gaussian 16 package^27^. The details of the procedure used to explore the configurational space of the dsDNA:**PNT** complexes are presented in Fig. 2 and the section Procedure S2 in the ESI.

From the semiempirical calculations the complexation enthalpies *H*_compl_ were calculated as:

1 $${H}_{{\text{compl}}}={H}_{{\text{dsDNA}}:{\text{PNT}}}^{{\text{OPT}}}-\left({H}_{{\text{dsDNA}}}^{{\text{OPT}}}+{H}_{{\text{PNT}}\_{\text{LE}}}^{{\text{OPT}}}\right)$$

where *H*_compl_ is the complexation enthalpy, while ${H}_{{\text{dsDNA}}:{\text{PNT}}}^{{\text{OPT}}}$, ${H}_{{\text{dsDNA}}}^{{\text{OPT}}}$ and ${H}_{{\text{PNT}}\_{\text{LE}}}^{{\text{OPT}}}$ are, respectively, the heats of formation of the dsDNA:**PNT** complex and of the isolated dsDNA and **PNT_LE** molecules, fully optimized with the PM7 method in water (COSMO).

From the DFT calculations, the complexation energies *E*_compl_ were computed according to a similar formula:

2 $${E}_{{\text{compl}}}={E}_{{\text{dsDNA}}:{\text{PNT}}}^{{\text{POPT}}}-\left({E}_{{\text{dsDNA}}}^{{\text{SP}}}+{E}_{{\text{PNT}}\_{\text{LE}}}^{{\text{OPT}}}\right)$$

where *E*_compl_ is the complexation energy, ${E}_{{\text{DNA}}:{\text{PNT}}}^{{\text{POPT}}}$ is the energy of partially optimized complex dsDNA:**PNT**, while ${E}_{{\text{dsDNA}}}^{{\text{SP}}}$—the energy obtained from the single point DFT calculations for the dsDNA structures obtained after the PM7 optimization, ${E}_{{\text{PNT}}\_{\text{LE}}}^{{\text{OPT}}}$—the DFT energy of the most stable conformer of ponatinib (**PNT_LE**) obtained in water (PCM).

## Results and discussion

### Voltammetric behavior of **PNT** on the BDD electrode

**Figure 3:**
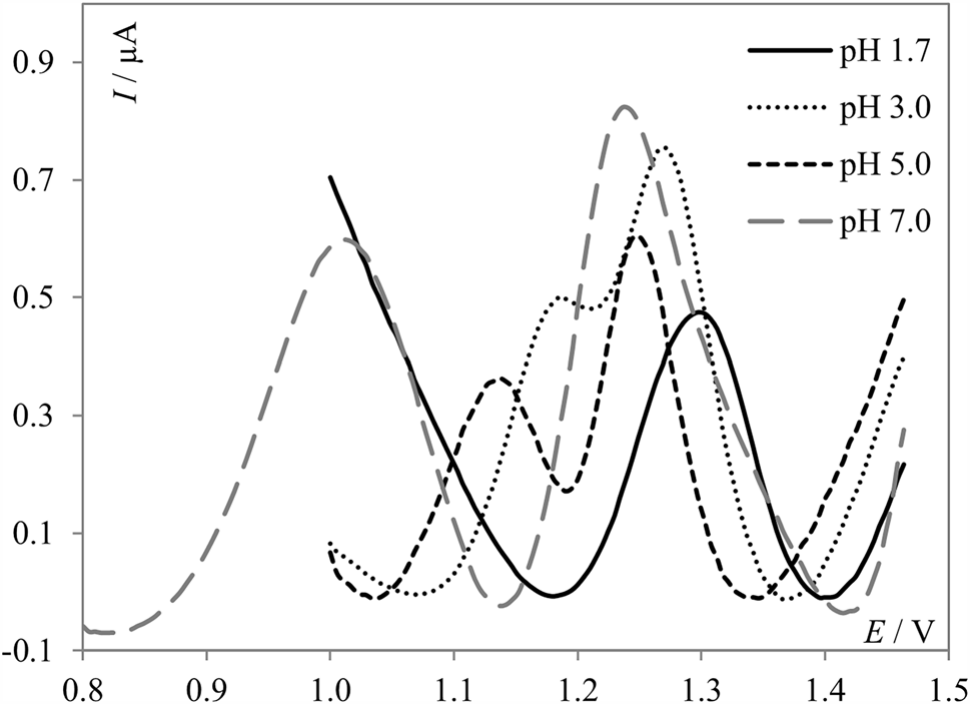
SW voltammograms of 5.0 × 10^–6^ mol L^−1^ **PNT** recorded in BR buffers at different pH values; Voltammograms are presented with background subtraction.

It is well known that the composition of the supporting electrolyte and its pH affect the observed electrode reaction and kinetics of the charge transfer process during voltammetric measurements. Thus, the pH-dependent oxidation process of **PNT** was initially studied in the wide pH range of 1.7–9.0 using BR buffers. As can be seen in Fig. 3, **PNT** signals were observed through the whole pH range. It was found that **PNT** provided one oxidation peak at a potential of approximately 1.3 V in a strong acidic medium. For higher pH values, a second signal appeared. As the acidity of the supporting electrolyte decreased, the peaks shifted toward less positive potential values, and the second peak became better separated. For pH 5.0 and higher, the separation of the two signals was sufficient for reliable measurements of the peak currents. For the first peak (the one of the right), the slope of the *E*_p_–pH dependence was 13 mV pH^−1^, showing that both protons and electrons are involved in the oxidation process of **PNT**, but it is difficult to determine the ratio between them. Such results indicated a complex electrode process presumably involving a multi-step reaction^28^. For the second peak (the one of the left), the slope of the same dependence was 51 mV pH^−1^, which is close to the theoretical value of 59 mV pH^−1^. This result suggests the participation of an equal number of protons and electrons in this part of the reaction.

Bearing in mind the sufficient separation of **PNT** signals for pH approximately 5.0 and higher, the influence of other buffers (such as acetate, phosphate, phosphate buffered saline; pH range of 4.5–8.0) on the analyte signals was also tested (data not shown). In all the tested buffers, the obtained peak currents and the separation of the signals were satisfactory and analogous to those obtained in the BR buffer. No significant improvement or deterioration of recorded signals was observed. Therefore, considering the fact that the main aim of the present work was DNA interaction studies, further measurements were performed in phosphate buffered saline at physiological pH and acetate buffer at pH 4.7. These buffers are commonly used for interaction studies^3,15,29^.

**Figure 4:**
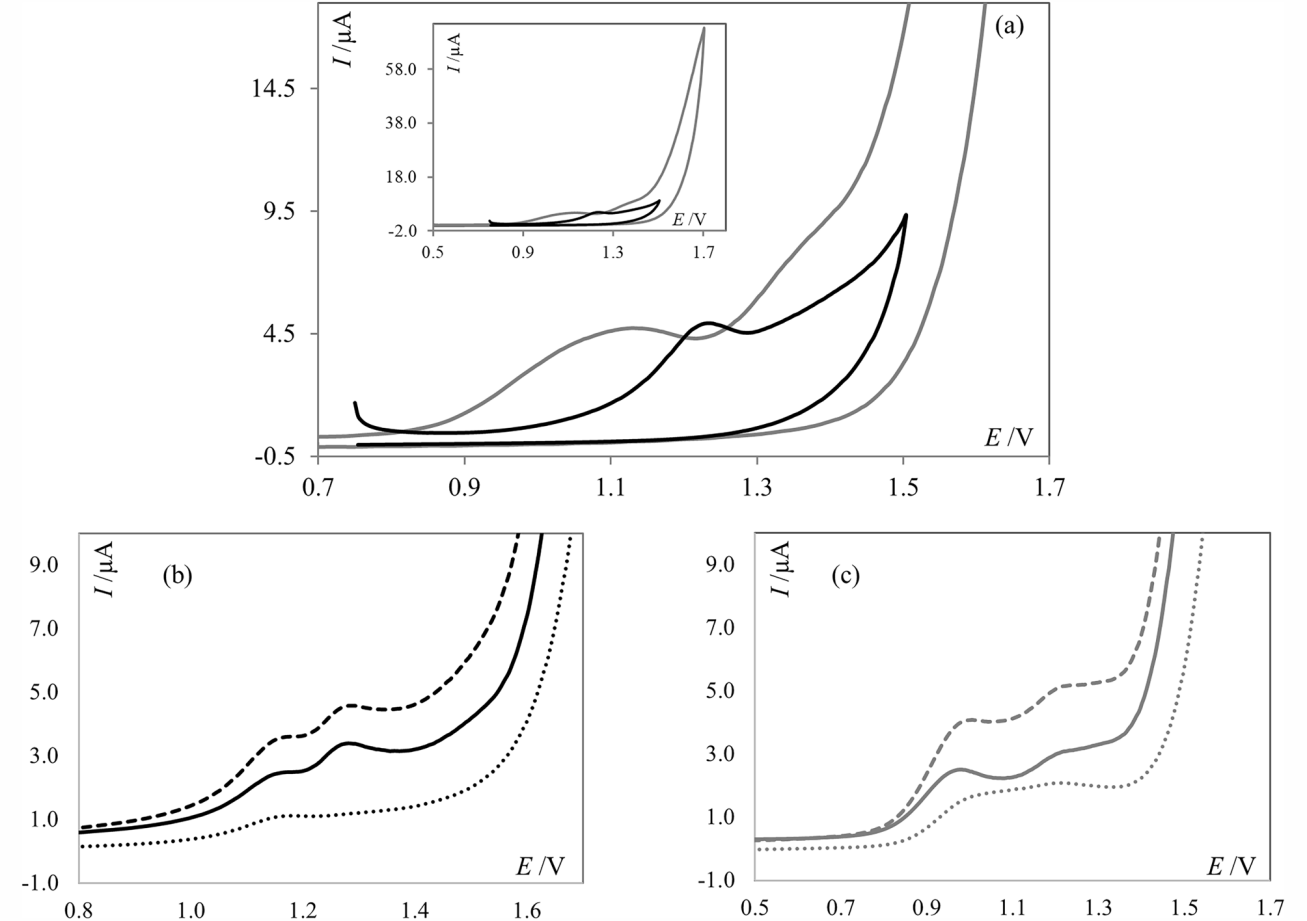
(**a**) Cyclic voltammograms of **PNT** (1.0 × 10^–4^ mol L^−1^), obtained at scan rate of 50 mV s^−1^, black line represents the voltammograms recorded in acetate buffer of pH 4.7, gray line in PBS of pH 7.4. Inset: Represents the same voltammograms but in the wider potential range; (**b**) SW voltammograms of **PNT** (1.0 × 10^−5^ mol L^−1^, net current—solid line) and the forward (dashed line) and backward (dotted line) components of **PNT** signals recorded in acetate buffer of pH 4.7; (**c**) SW voltammograms of **PNT** (1.0 × 10^−5^ mol L^−1^, net current—solid line) and the forward (dashed line) and backward (dotted line) components of **PNT** signals recorded in PBS of pH 7.4.

In the next experimental step, cyclic voltammetry was applied to gain insight into the electrochemical oxidation process of **PNT** using both chosen supporting electrolytes, i.e. PBS and acetate buffer. As can be seen in Fig. 4a **PNT** exhibited either two or one anodic peaks, depending on the pH of the supporting electrolyte. In PBS two well-separated signals were observed, which are in excellent agreement to the SWV voltammograms. In acetate buffer, only one signal was observed ca. 1.2 V, suggesting that during the measurement, two signals visible in square wave experiments overlapped. Based on the cyclic voltammograms it may be also observed that in the reverse scan, no corresponding cathodic signals were visible. Thus, it may be concluded that the oxidation of ponatinib on BDD electrode is an irreversible process. This was also confirmed by using the SW voltammetry, in which the observed forward and backward components of **PNT** signals tipped in the same direction for both buffers (Fig. 4b and c), thus proving the irreversible character of the electrochemical oxidation of **PNT**.

In order to check whether the oxidation of **PNT** is a diffusion- or adsorption-controlled process the scan rate studies were performed. The measurements were carried out within the scan rate (*v*) range from 50 to 500 mV s^−1^. A linear dependence between the peak currents (*I*_p_) and scan rates were observed in the case of both supporting electrolytes, suggesting adsorption-controlled nature of the **PNT** electrode processes. This evidence was further verified with construction of the logarithmic plot *I*_p_ vs. log *v*. The log *I*_p_–log *v* slopes were found to be 0.71 and 0.97 for PBS and acetate buffer, respectively, where values close to 1.0 are specific for processes controlled by adsorption^14,30^. Based on these results, it can be stated that electrochemical process in the acetate buffer is controlled by adsorption, whereas in phosphate buffered saline, a mixed diffusion-adsorption controlled process was observed.

### Electrochemical studies of **PNT**-dsDNA interaction

**Figure 5:**
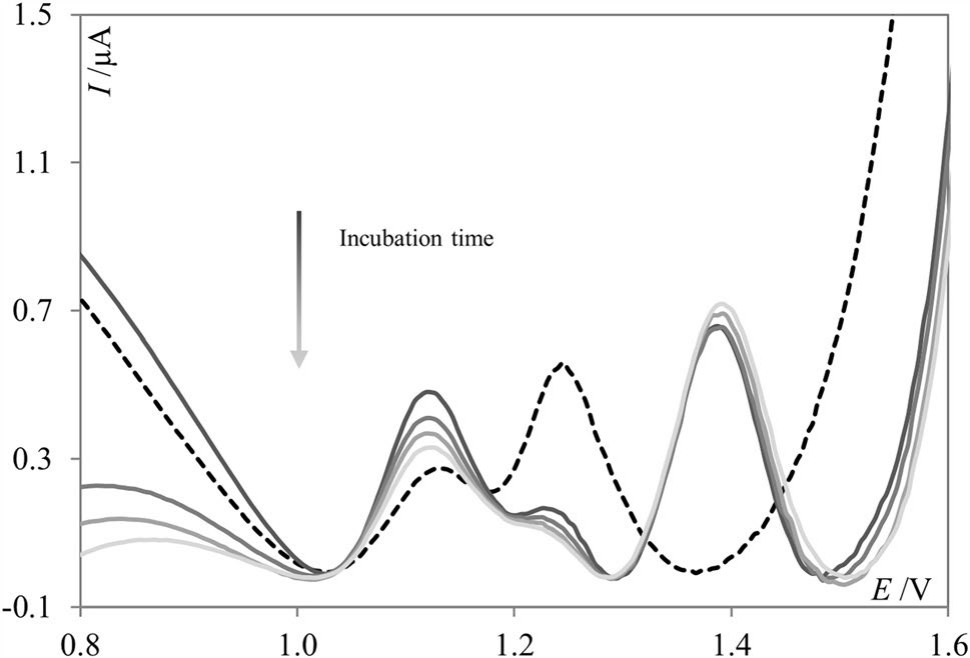
SW voltammograms of **PNT** (dashed, black line) and **PNT** in the presence of dsDNA (solid, gray lines), after different incubation periods (10, 30, 60 and 90 min) recorded in acetate buffer of pH 4.7; *c*_**PNT**_ = 5.0 µmol L^−1^, *c*_dsDNA_ = 80 mg L^−1^.

**Figure 6:**
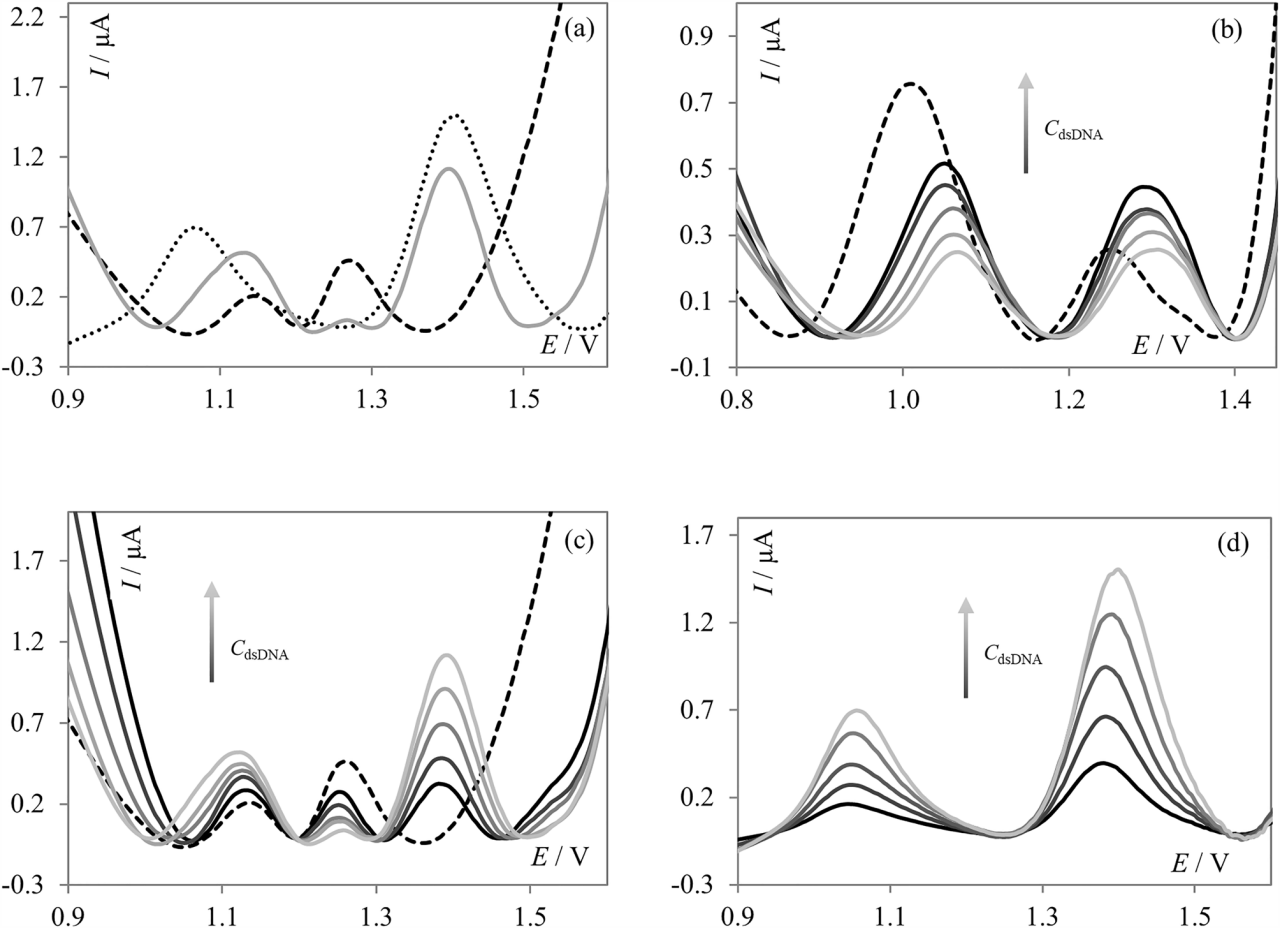
(**a**) SW voltammograms of **PNT** (dashed, black line), **PNT** in the presence of dsDNA (solid, gray line), and dsDNA (dotted, black line) recorded in acetate buffer of pH 4.7; *c*_**PNT**_ = 5.0 µmol L^−1^, *c*_dsDNA_ = 80 mg L^−1^; (**b**) SW voltammograms of **PNT** (dashed, black line) recorded in PBS of pH 7.4, in the presence of an increasing amount of dsDNA (solid, gray lines), *c*_**PNT**_ = 5.0 µmol L^−1^, *c*_dsDNA_ = 10—80 mg L^−1^; (**c**) SW voltammograms of **PNT** (dashed, black line) recorded in acetate buffer of pH 4.7, in the presence of increasing amount of dsDNA (solid, gray lines), *c*_**PNT**_ = 5.0 µmol L^−1^, *c*_dsDNA_ = 10–80 mg L^−1^; and (**d**) SW voltammograms of dsDNA and its increasing concentration recorded in acetate buffer of pH 4.7, *c*_dsDNA_ = 10–80 mg L^−1^.

The studies of the interaction between **PNT** and DNA were initially performed by incubating a solution of 5.0 µmol L^−1^ **PNT** with 80 mg L^−1^ of dsDNA. Both supporting electrolytes, i.e., acetate buffer of pH 4.7 and PBS of pH 7.4 were used for that purpose; however, the results obtained in PBS were insufficiently repeatable to draw reliable conclusions. Thus, the following results are the outcome of interaction studies performed via the incubation procedure in acetate buffer. The SW voltammograms were recorded after different times of incubation (10, 30, 60, and 90 min). The surface of the boron doped diamond electrode was renewed between every measurement to avoid the blockage of the electrochemically active electrode surface via adsorption of the oxidation products of **PNT** and/or dsDNA. After the addition of dsDNA to the voltammetric cell with **PNT**, significant changes in recorded voltammograms were observed, and the extension of incubation time deepened the changes (Fig. 5). As can be seen in Fig. 5, **PNT** exhibited two signals, the left one at approximately 1.15 V and the right one at approximately. 1.25 V (dashed line). After 10 min upon the addition of dsDNA, the left signal significantly increased, the right one decreased, and one additional signal at approximately 1.4 V appeared. The left signal, after the initial increase, gradually decreased with the incubation time, while the right signal (approximately 1.25 V) current progressively decreased in a time-dependent manner. It is worth noting here that dsDNA is an electrochemically active compound, and in the applied potential range two signals are expected^31^, one corresponding to the oxidation of deoxyguanosine (dGua) at a lower potential value, and the second one to the oxidation of deoxyadenosine (dAdo) at a higher potential. In the present work, the electrochemical behavior of dsDNA was recorded as a control experiment, and two signals were observed at approximately 1.05 V and 1.4 V (Fig. 6a, dotted line). Thus, based on the results shown in the Fig. 5, it may be stated that the peak current at approximately 1.15 V initially increased after the short incubation with dsDNA due to some conformational changes in **PNT** molecules, which initially facilitated the oxidation of the drug by exposing the group responsible for oxidation. The further gradual decrease of the oxidation peak indicated a preferential interaction between **PNT** and dGua residues in the dsDNA. It can also presumed that the signal at 1.4 V corresponds to the oxidation of dAdo. As no significant changes occurred with the signal at approximately 1.4 V during the incubation, it may be presumed that the presence of **PNT** did not affect the oxidation of deoxyadenosine, and condensation or aggregation of DNA strands was not observed^32^. During the evaluation of **PNT**-dsDNA interaction in the incubated solution, no significant shifts of the peak potentials were observed. As a shift of the peak potential is commonly observed in intercalative types of interaction, we can rather exclude this type of interaction between **PNT** and DNA. What is more, DNA oxidative damage was not detected, as oxidation signals of 8-oxoguanine or 2,8-dihydroxyadenine^32^ were not observed, which means that **PNT** did not induce oxidative damage to double-stranded DNA.

Next, the SW voltammograms of **PNT** (at its fixed concentration) in the absence and presence of increasing amount of dsDNA were recorded in an acetate buffer of pH 4.7 and PBS of 7.4; the obtained results were illustrated in Fig. 6. In Fig. 6d, SW voltammograms of dsDNA and its increasing amount in acetate buffer were shown for comparison purposes. In these experiments, the signals were recorded immediately after the addition of dsDNA to the **PNT** solution. The voltammograms recorded in the acetate buffer of pH 4.7 (Fig. 6c) significantly differed from the voltammograms obtained in PBS of pH 7.4 (Fig. 6b). In the second case, both observed signals gradually decreased upon the addition of increasing amounts of dsDNA. What is more, both signals slightly shifted toward more positive potential values when compared to **PNT** signals. In acetate buffer, the right signal increased whereas the left decreased upon the addition of increasing amounts of dsDNA. Moreover, one additional signal appeared at approximately 1.4 V, and its peak current gradually increased upon the addition of dsDNA. In the acetate buffer, the signal shift was so slight as to be insignificant. In conclusion, both supporting electrolytes allowed us to observe the occurring interaction; however, based on the obtained results, it can be concluded that the pH of the supporting electrolyte strongly affected the direction of the interaction. In general, **PNT** induced conformational modifications in dsDNA molecules^33^. The decrease of the signals and the shift observed in PBS may be correlated with DNA double helix condensation and **PNT** intercalation^33^. However, the observed shift in the peak potential is much smaller than shifts described by other authors for intercalation^34^, making it rather difficult to definitively identify the observed interaction type. As signal decrease was observed in the case of both signals in PBS, and no additional signal appeared, it can be concluded that **PNT** interacts with both dGua and dAdo at physiological pH. Whereas the dependencies observed in the acetate buffer strongly suggested the interaction between **PNT** and guanine residues in the dsDNA^17^, which is consistent with the results obtained in the incubation procedure. In the acidic environment, as no significant shift in the peak potential was observed, it can be stated that intercalation likely did not occur.

### Results of the DFT calculations

More detailed information on the structural properties of the tested compounds, as well as on the stability of the dsDNA:**PNT** complexes and the intermolecular interactions occurring within them, can be obtained using theoretical chemistry methods. Since the electrochemical measurements were performed in an aqueous solution, the solvent effect was also incorporated into the quantum chemical calculations using implicit models.

**Figure 7:**
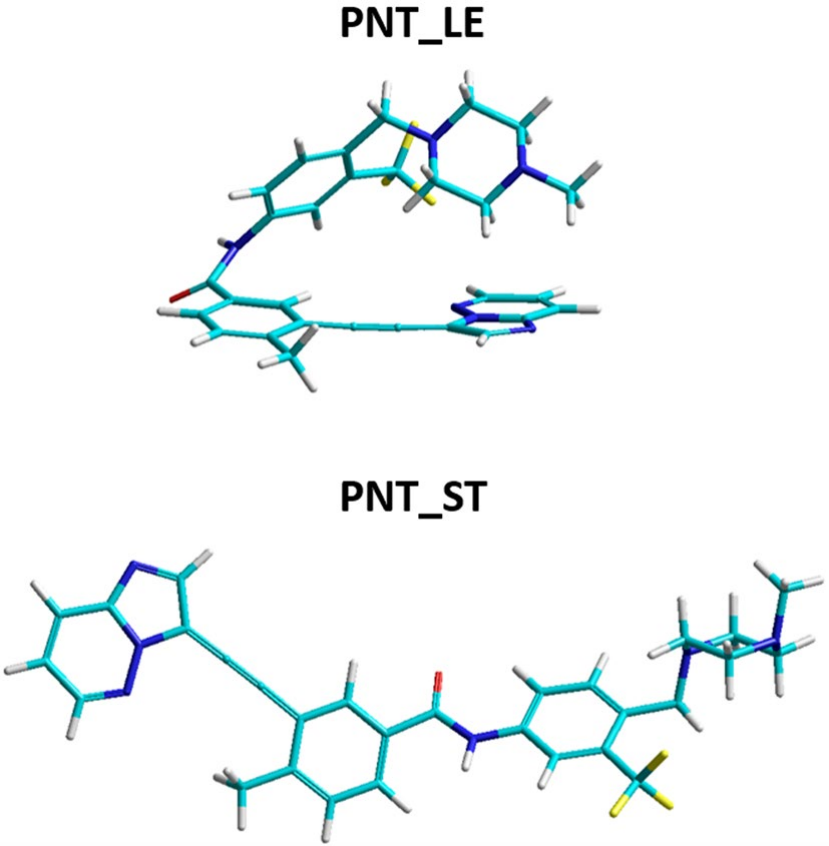
The most stable structure of ponatinib (**PNT_LE**) and its stretched conformer (**PNT_ST**) obtained from the M062X-GD3/6-31G(d,p) calculations in water (PCM). Atom colors: carbon—cyan, nitrogen—dark blue, fluorine—yellow, oxygen—red, hydrogen—grey.

First, the properties of **PNT** in water were explored through an extensive conformational search. The final M062X-GD3/6-31G(d,p)/PCM results allowed the identification of the most stable structure of **PNT**, but at the same time revealed that the energy of other conformers is not much higher, so they may also be present in the solution. Therefore, for further studies on complexes of dsDNA:**PNT**, two different **PNT** structures were selected, both of which are shown in Fig. 7. The first one, **PNT_LE**, is the lowest energy conformer in water as indicated by the results of DFT calculations. It has a twisted structure, with the methylpiperazine group located above the two aromatic rings. The second, **PNT_ST**, is the first fully stretched structure found among all tested conformers ordered by increasing energy. It is worth mentioning that, in this order, **PNT_ST** is numbered 157, and its total energy is higher than that of **PNT_LE** by only 8.4 kcal mol^−1^. Thus, within this relatively narrow energy window, there are many other intermediate structures between **PNT_LE** and **PNT_ST**.

**Figure 8:**
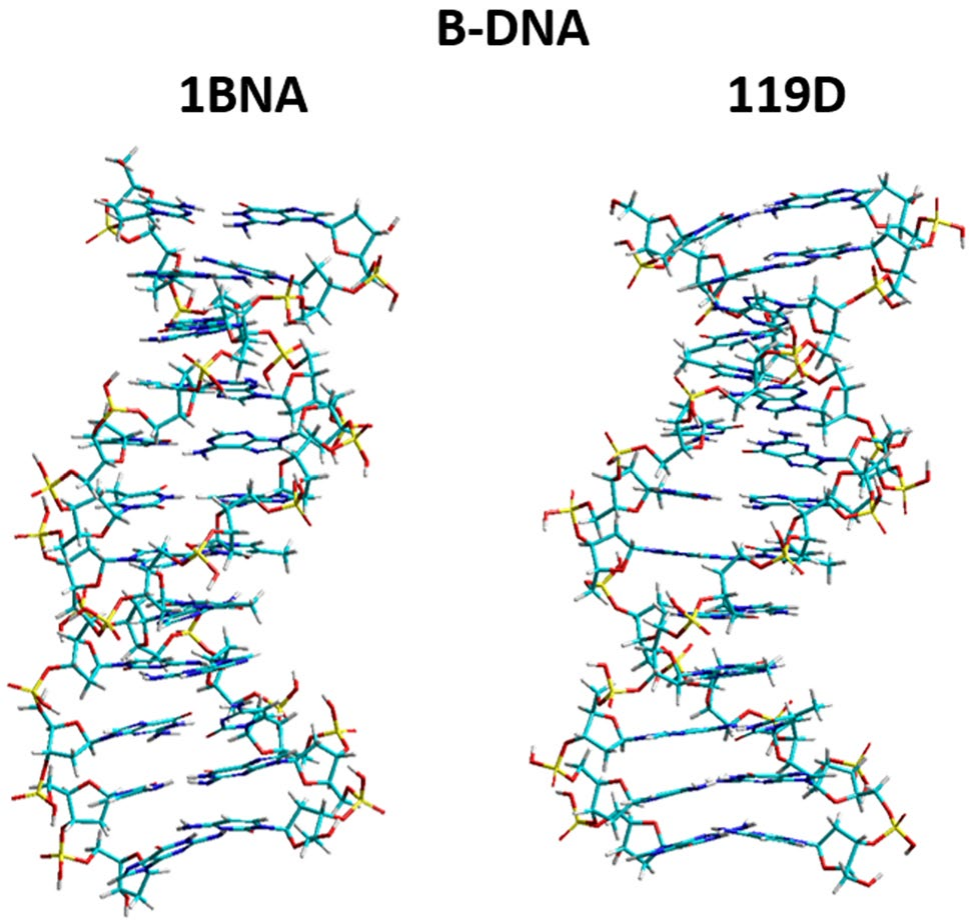
Structures of the two B-DNA double-stranded dodecamers 1BNA and 119D obtained after the optimization of the crystal structures with the semiempircal PM7 method in water (COSMO) and the MOZYME module. Atom colors: carbon—cyan, nitrogen—dark blue, phosphorus—yellow, oxygen—red, hydrogen—grey.

The two dodecamers (double-helix), based on the crystal structures (1BNA and 119D) and optimized with the semiempirical method PM7 in water, which were used to create initial models of the dsDNA:**PNT** complexes, are shown in Fig. 8. As can be seen, due to the sequence differences, the two strands of B-DNA in the model 1BNA appear to be closer than in 119D, which may have some effect on stability of the dsDNA:**PNT** complexes.

**Figure 9:**
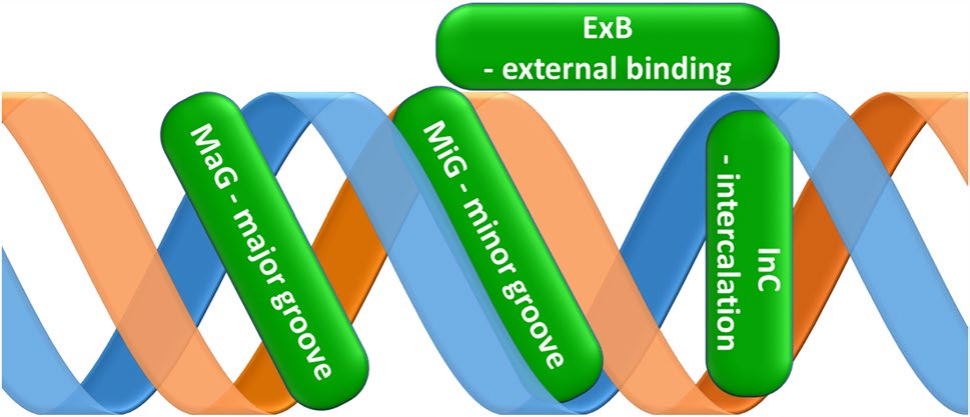
Schematic picture of the typical binding sites in dsDNA.

**Figure 10:**
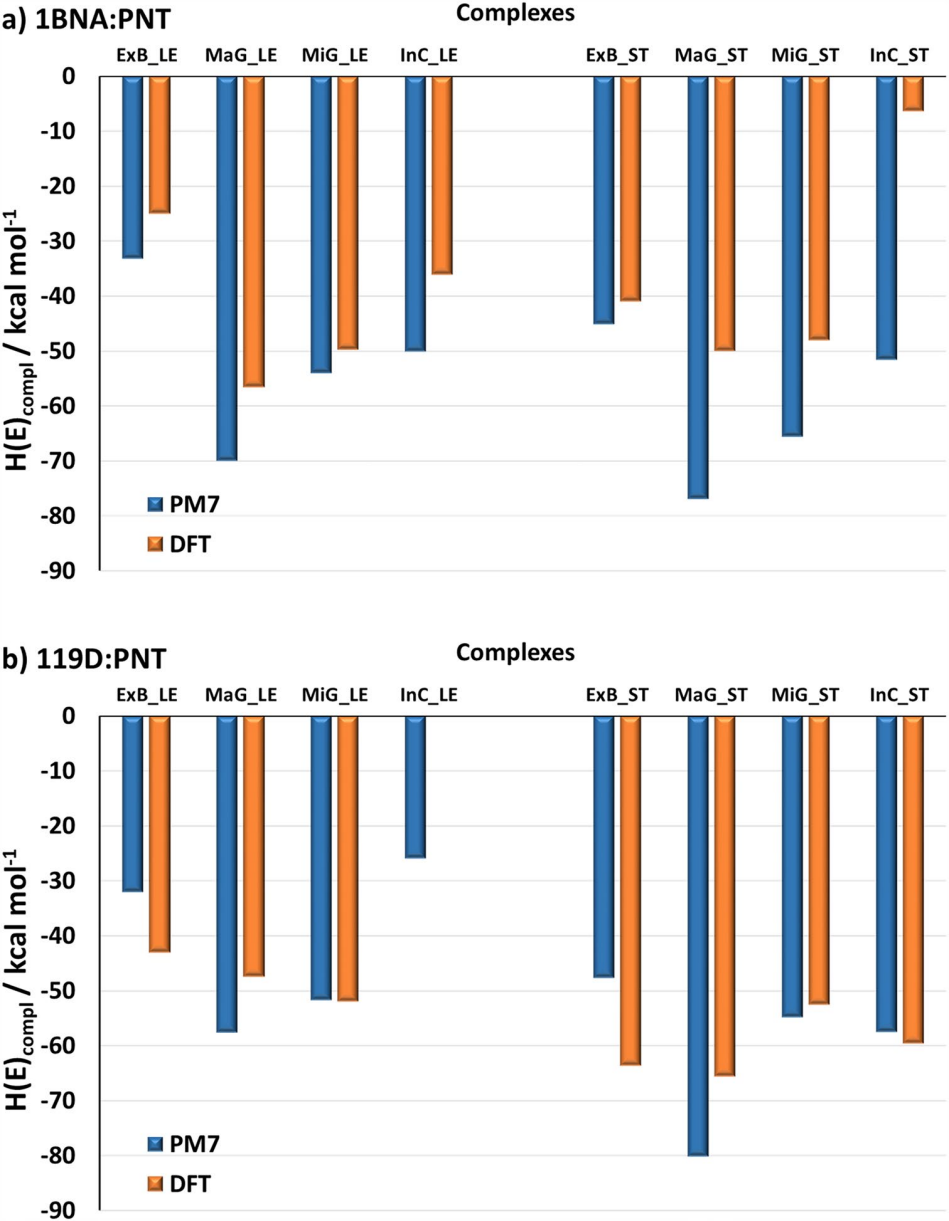
Comparison of the complexation PM7 enthalpies *H*_*compl*_ and DFT energies *E*_compl_, obtained for the complexes 1BNA:**PNT** (**a**) and 119D:**PNT** (**b**), presented in Figs. S3–S6 in the ESI. The PM7 heats of formation and DFT total energies obtained for PNT, dsDNA and all complexes are given in Table S1 in the ESI.

**Figure 11:**
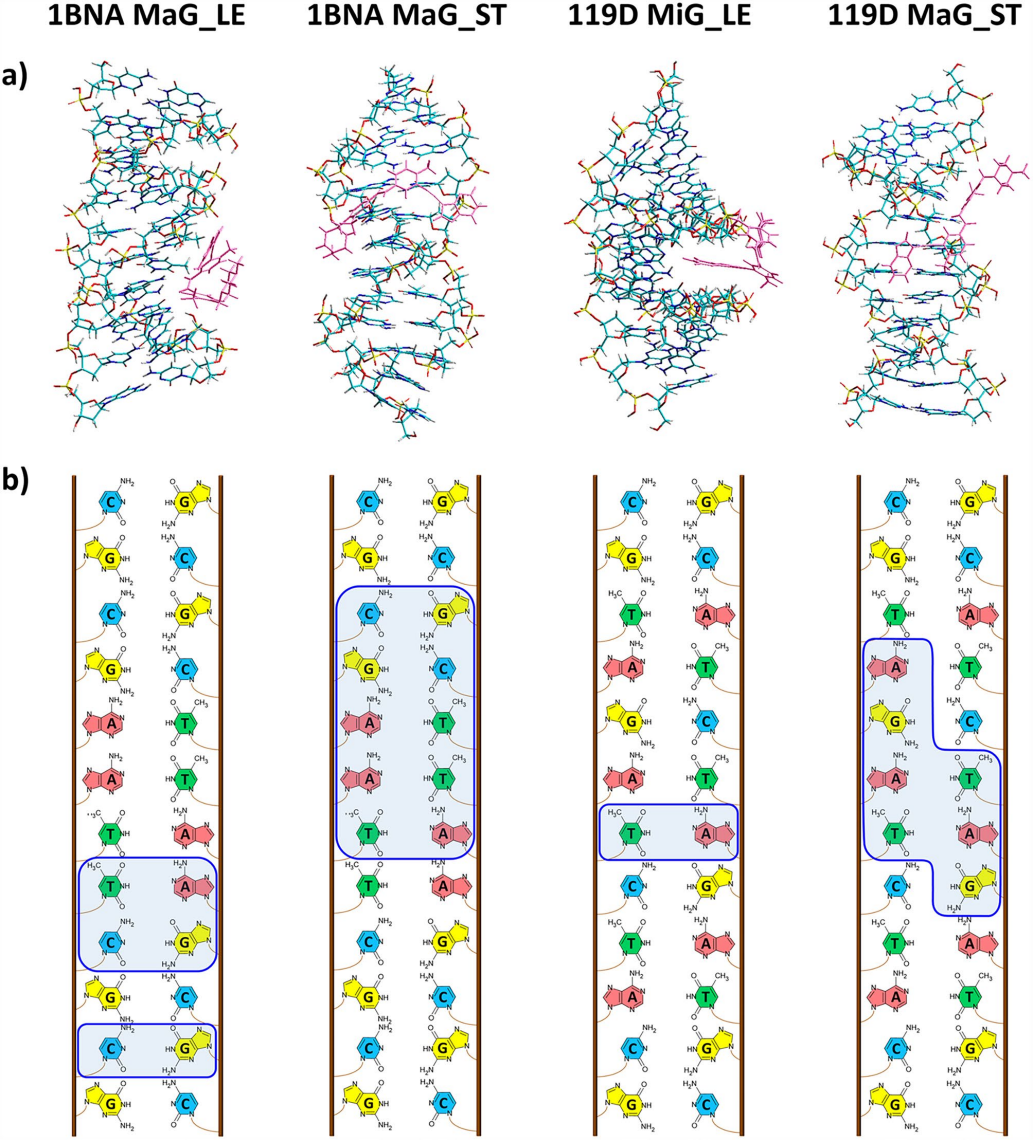
Structures of the most stable configurations obtained from the DFT calculations for the four different models of the dsDNA:**PNT** complexes (**a**) and the corresponding schematic images of the dsDNA sequence, on which the nucleobases closest to **PNT** are outlined with a blue line (**b**). In (**a**), the dsDNA atom colors are the same as in Fig. 8, while the **PNT** molecule is colored pink. Nucleobases symbols in (**b**): A—adenine, G—guanine, C—cytosine, T—thymine.

The four sites in dsDNA considered for **PNT** binding are depicted in Fig. 9. The final PM7 complexation enthalpies (*H*_compl_) and DFT complexation energies (*E*_compl_) obtained at these sites with the two dsDNA models and two **PNT** conformers are compared in Fig. 10. The most stable structures, as indicated by the DFT results for the four models of the dsDNA:**PNT** complex, are presented in Fig. 11a, while all structures corresponding to the results shown in Fig. 10, are displayed in Figs. S3–S6 in the ESI.

As can be seen, the results obtained with both PM7 and DFT methods indicate that in all four sites the **PNT** molecule forms stable complexes with dsDNA, regardless of the DNA model used. For a given B-DNA sequence and a specific **PNT** conformer, the trends in the PM7 enthalpies *H*_compl_ and the DFT complexation energies *E*_compl_ for the different interaction sites are similar, but the *H*_compl_ values are significantly more negative than the corresponding *E*_compl_ values.

According to the PM7 results, for both dsDNA models, the conformer **PNT_ST** is more strongly bound in all four sites than **PNT_LE**. A similar tendency is observed in the DFT complexation energies for 119D:**PNT**, while for 1BNA:**PNT** the trend is the opposite, except for ExB_ST, for which *E*_compl_ is lower than that for ExB_LE. Thus, the DFT results suggest that the binding of **PNT** by dsDNA depends on the order of nucleotides in the DNA. In the case of 1BNA:**PNT**, the major and minor grooves are preferred sites for the complexation of both **PNT_LE** and **PNT_ST** conformers. The most negative *E*_compl_ of − 56.6 kcal mol^−1^ is obtained for the MaG_LE complex, but for the other sites it differs by only a few kilocalories per mole. An exception is the InC_ST configuration, for which the complexation energy is only − 6.5 kcal mol^−1^. In contrast, this particular configuration appears to be very stable in the case of 119D:**PNT**, where it has the complexation energy of − 59.7 kcal mol^−1^. As already mentioned, the stretched structure of **PNT** in general fits much better to the 119D double helix; the most stable configuration for 119D:**PNT** is MaG_ST with the complexation energy of -65.6 kcal mol^−1^. The significant difference between the InC_ST complexes formed by **PNT_ST** with the two dsDNA models may be due to the fact that in 1BNA the two strands are more tightly coiled than in 119D, so intercalation of **PNT** into 1BNA causes stronger deformation of the latter. Among the complexes of 119D, a surprisingly small *E*_compl_ (-0.2 kcal mol^−1^) was obtained for the InC_LE configuration. In this case, steric hindrance may be responsible for so small stabilization of this complex.

**Figure 12:**
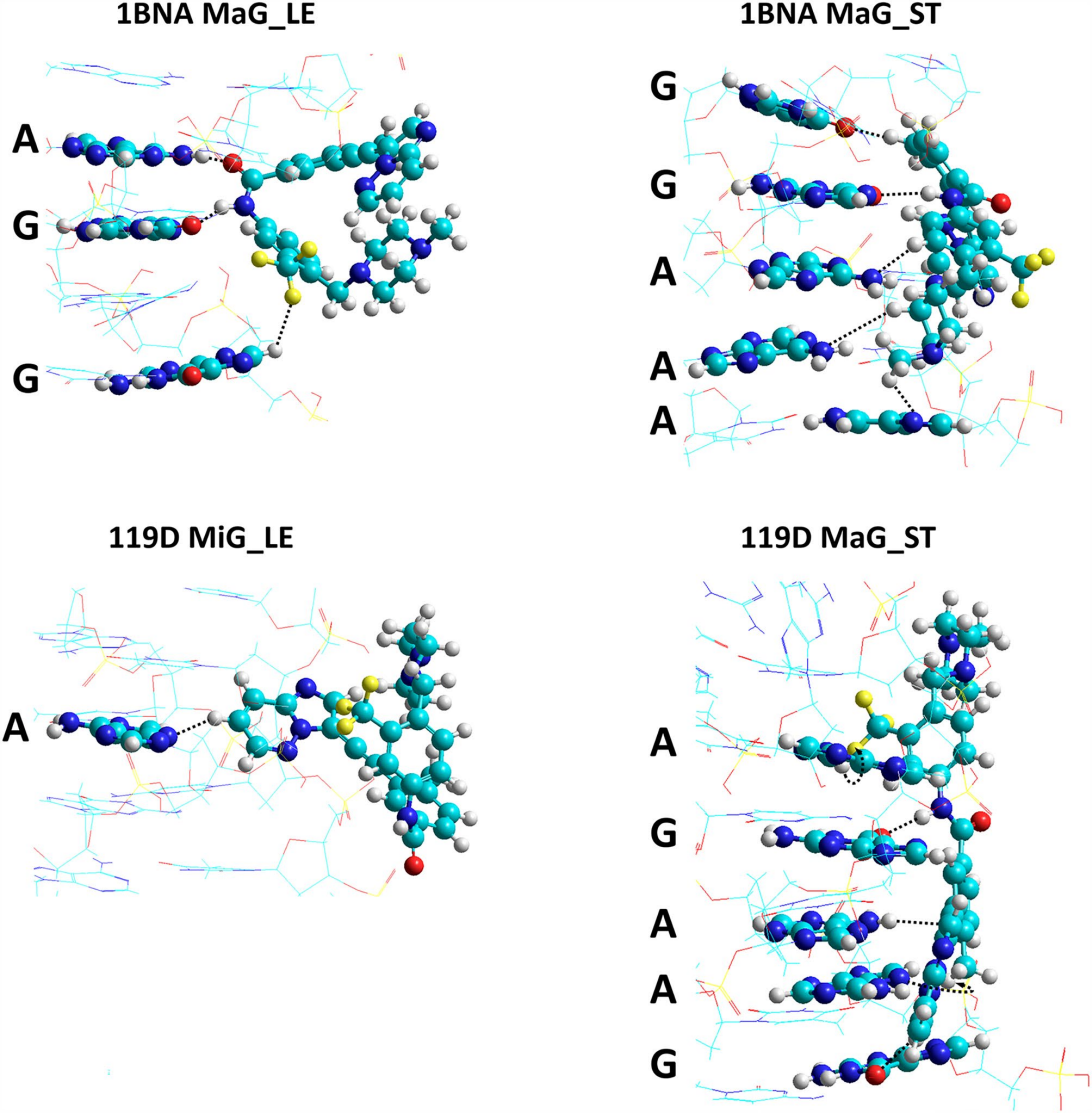
Magnification of fragments of the most stable complexes dsDNA:**PNT** shown in Fig. 11; the **PNT** molecule (always on the right) and the nearest adenine (A) and guanine (G) are rendered with spheres and cylinders. Atom colors are the same as in Figs. 7 and 8. The black dashed lines connect the closest atoms in each base and **PNT**.

A closer inspection of the most stable structures indicated for each dsDNA:**PNT** model by the DFT results (Fig. 11a) allows for the identification of the nucleobases that directly interact with **PNT** (Fig. 11b). The specific arrangement of the two electrochemically detectable nucleobases, guanine and adenine, is illustrated in Fig. 12. **PNT** is a relatively large molecule, and as a result, it can interact with several nucleobases. As expected, for the stretched conformer **PNT_ST**, there are more such interactions. In the case of guanine, the interaction most often occurs via its oxygen atom, which is usually involved in hydrogen bonding with the H-N or H-C bonds of **PNT**. The O…H distances in the O…H–N hydrogen bonds range from 1.96 (upper guanine in 119D MaG_ST in Fig. 12) to 2.37 Å (upper guanine in 1BNA MaG_LE), while in the O…H–C hydrogen bonds: from 2.32 (lower guanine in 119D MaG_ST) to 2.42 Å (upper guanine in 1BNA MaG_ST). According to the Jeffrey’s categorization^35,36^, these are moderate or weak hydrogen bonds but, considering their number, they can be quite important and may have some effect on the electrochemical activity of dsDNA. Adenine forms various hydrogen bonds, but their geometrical parameters (e.g. longer H…X distances) suggest that they are weaker than those formed by guanine. The most prominent hydrogen bond observed for adenine is the one in the complex 1BNA MaG_LE, between its NH_2_ hydrogen atom and the oxygen atom in **PNT** (the H…O distance is 2.20 Å). In other configurations, they are formed between one of the nitrogen atoms in adenine and H-C bonds in **PNT**. Of course, apart from hydrogen bonds, other interactions between dsDNA and **PNT** (such as electrostatic and dispersion forces) also contribute to the stability of the complex.

## Conclusion

The interaction of **PNT**, a third-generation tyrosine kinase inhibitor, with double-stranded DNA was investigated for the first time using electrochemical and computational techniques. The voltammetric studies were conducted using two approaches. The first approach involved experiments performed in an incubated solution, where both the concentration of the analyte and DNA were fixed, and different incubation times were tested. The second approach relied on measurements performed in a solution containing **PNT** and dsDNA in different concentration ratios. Based on the voltammetric results, obtained at physiological pH, it was observed that **PNT** interacted with both dGua and dAdo residues in dsDNA molecules. In an acidic environment, voltammetric data suggest that **PNT** interacts mainly with dGua residues. It is known that evaluating the type of interaction with electrochemical techniques should be confirmed with other methods, as they only allow for the formulation of a hypothesis based on the peak potential shift. In this study, conclusions drawn from the peak potential shift may be summarized as follows: in the acidic environment, since no shift in the peak potential was observed, it can be concluded that intercalation likely did not occur. Hence, groove binding is suggested to be more prevalent. At physiological pH, a shift in the peak potential value was observed; however, it was so insignificant that it was not possible to draw a definitive conclusion about the ongoing interaction type.

The results of the theoretical research confirm that groove binding of PNT is energetically more favorable. According to the semiempirical PM7 results, the stretched structure **PNT_ST** is, in general, more strongly bound than its most stable structure **PNT_LE** and in both forms of dsDNA the major groove is the preferred site for the complexation of **PNT**. The complexation enthalpies corresponding to the MaG_ST configurations with the 1BNA and 119D models of dsDNA are − 77 and − 80 kcal mol^−1^, respectively. The absolute values of the DFT complexation energies are smaller than those obtained from the PM7 calculations and suggest that the stability of complexes may depend on both the structure of **PNT** and the order of nucleotides in DNA. For both dsDNA models, the MaG site is favored, but in the case of 1BNA the complex with **PNT_LE** is the most stable (*E*_*compl*_ = − 57 kcal mol^−1^), while for 119D the binding of **PNT_ST** is energetically more profitable (*E*_compl_ = − 66 kcal mol^−1^). Nevertheless, given the relatively minor differences in *E*_compl_ for some of the other alternative configurations, it is likely that these configurations will also form in parallel under certain conditions. Analysis of the structures of the complexes obtained from quantum-chemical calculations showed that both electrochemically detectable nucleobases, adenine and guanine, form hydrogen bonds with the drug. The geometric characteristics of these bonds suggest that those formed with guanine are stronger than those formed with adenine.

In general, based on both voltammetric and computational results it can be concluded that an intercalative type of interaction can likely be excluded, whereas major groove binding is more probable to occur. Both approaches also suggest that **PNT** interacts with guanine and adenine residues in dsDNA molecules, with a preference toward guanine.

### Supplementary Information

Supplementary Information.

## Data Availability

The datasets used and/or analysed during the current study available from the corresponding author on reasonable request.
