# Supplementary material for: Electrochemical and theoretical studies of the interaction between anticancer drug ponatinib and dsDNA
Source: Sci Rep. 2024 Jan 27;14:2278. doi: 10.1038/s41598-024-52609-z (PMC10821894; doi:10.1038/s41598-024-52609-z)
Supplement: Supplementary file 1 — Supplementary Information. [file 41598_2024_52609_MOESM1_ESM.docx]

# **Electrochemical and theoretical studies of the interaction between anticancer drug ponatinib and dsDNA**

# Sylwia Smarzewska^a^, Anna Ignaczak*^b^, Kamila Koszelska*^a^

^a^ University of Lodz, Department of Inorganic and Analytical Chemistry, 12 Tamka Str, 91-403 Lodz, Poland

^b^ University of Lodz, Department of Physical Chemistry, 163/165 Pomorska Str, 90-236 Lodz, Poland

**Electronic Supporting Information**

**Table of Contents:**

**Figure S1** – Flow chart of the procedure applied to find the lowest energy structures of ponatinib S3

**Figure S2**  – Initial structure and torsion angles varied in the conformational search for PNT S4

**Procedure S1** – Description of the conformational search performed for PNT S5

**Procedure S2** – Description of the calculations performed for the dsDNA:PNT complexes S6

**Figure S3** – Structures of dsDNA:PNT_LE obtained from the PM7 calculationsS7

**Figure S4** – Structures of dsDNA:PNT_ST obtained from the PM7 calculationsS8

**Figure S5** – Structures of dsDNA:PNT_LE obtained from the DFT calculationsS9

**Figure S6** – Structures of dsDNA:PNT_ST obtained from the DFT calculationsS10

**Table S1** – PM7 heats of formation and DFT energies for PNT, dsDNA and dsDNA:PNT S11

**References** S12

**
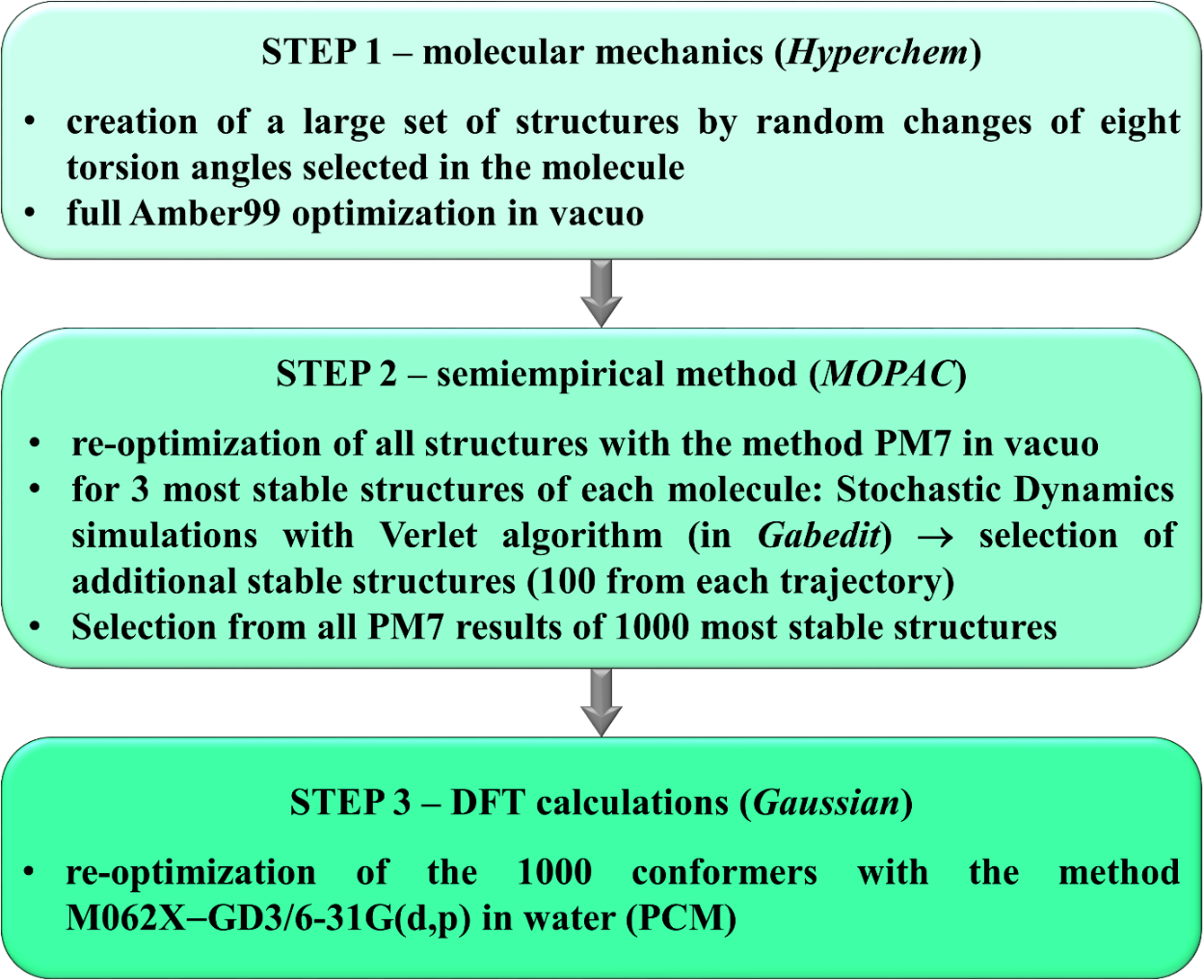
**

**Figure S1.** Flow chart of the procedure used in the search for the lowest energy conformers of ponatinib (PNT).

**
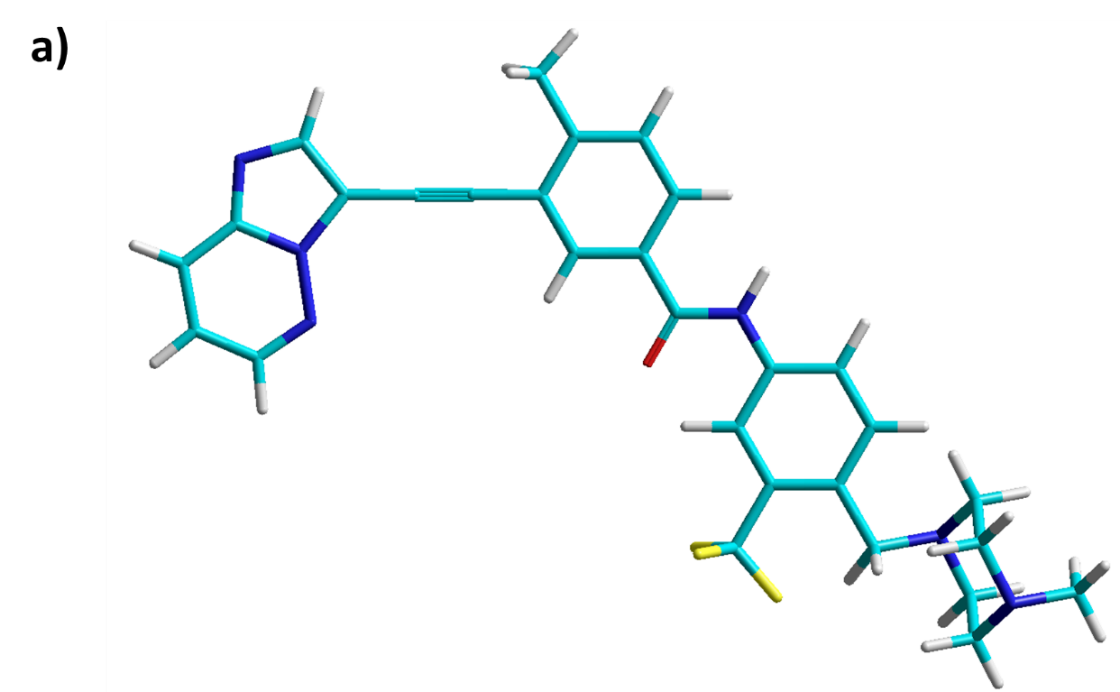
**

**
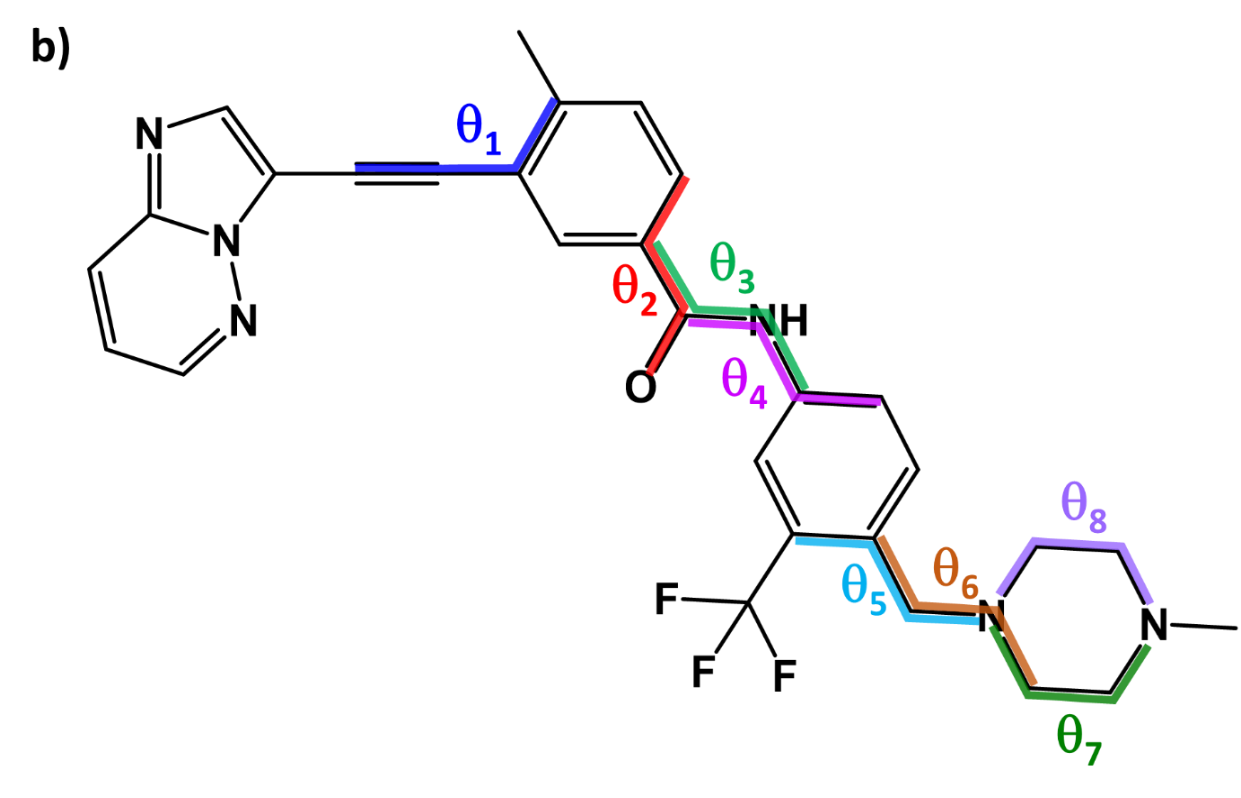
**

**Figure S2.** The initial structure of the PNT molecule (a) and the torsion angles varied (b) in the conformational search performed in the Hyperchem program. Atom colors in a): carbon – cyan, nitrogen – dark blue, fluorine – yellow, oxygen – red, hydrogen –grey.

**Procedure S1**

*Detailed description of the conformational search performed for the PNT molecule*

**Step 1**: The initial structure of PNT (Figure S1a) was built in the HyperChem program [1]. The test conformers were generated using the Amber99 molecular mechanics force fields and the Conformational Search module available in the HyperChem program. In the initial structure eight torsion angles were selected (Figure S1b). Different conformers were created by randomly and simultaneously varying the acyclic bond dihedral angles θ_1_-θ_6_ in the range of 0-180° and of the angles θ_7_ and θ_8_ in the piperazine ring. Each new structure was optimized. After the automatic removal of duplicates, the lowest energy conformers, having relative energies below 100 kcal mol^-1^, were stored. The resulting 12258 structures served as initial geometries for the next stage.

**Step 2**: All of these conformers were subsequently optimized in vacuo using the semiempirical method PM7 available in the MOPAC program[2]. From all the geometries obtained, three structures corresponding to the lowest heats of formation were selected. For each of these, a simulation was performed using the Molecular Dynamics Conformational Search module available in the Gabedit program [3] and the MOPAC program. The goal of this stage was to explore whether the molecule can adopt lower energy geometries. The simulations were performed using Stochastic Dynamics via the Verlet algorithm. To overcome possible higher energy barriers, the simulations were conducted at a temperature of T=1000 K for a period of 10 ps with a time-step of 1 fs. In each simulation, 100 lowest energy conformations from the calculated trajectory were selected and subsequently optimized with the PM7 method. The simulations were repeated, starting from the most stable conformer selected in a given simulation, until no lower energy structure was found. The conformers obtained from the initial PM7 optimization and those selected after the simulations were merged. From this set, the 1000 lowest energy conformers were selected and used as initial geometries for the DFT calculations.

**Step 3**: The structures selected after Step 2 were fully optimized using the Gaussian 16 program [4]. The optimization was performed with the 6-31G(d,p) basis set and the M062X-GD3 method, which is the hybrid metafunctional M06-2X [5] with the Grimme empirical pairwise long range (dispersion) corrections GD3 [6]. The DFT calculations were conducted in water, described by the Polarizable Continuum Model (PCM) [7].

**Procedure S2**

*Detailed description of the calculations performed for each position of PNT in the dsDNA:PNT complexes*

**Step 1**: In the initial structures of complexes, the DNA double helix was placed in the center of the workspace in HyperChem. For external binding (ExB), major groove (MaG) and minor groove (MiG), the PNT molecule (either PNT_LE or PNT_ST conformer) was placed at two slightly different positions corresponding to a given site. It was then rotated in steps of 30° around each axis in the ranges: 0-180° (X), 0-360° (Y) and 0-360° (Z). This yielded 864 different structures for a single position, thus 1728 structures for each site. For intercalation (InC), test structures were created by manually inserting the drug between the nucleotides at six different positions along the DNA double helix. At each position, different orientations of PNT were tested: two for PNT_LE and four for PNT_ST. For PNT_LE, due to its compact structure and insufficient space in dsDNA, in some cases, it was necessary to first adjust slightly the dsDNA structure to the presence of the drug by performing up to 60 initial steps of optimization of dsDNA with the PNT structure frozen.

**Step 2**: All structures created in Step 1 were fully optimized (no constraints) using the semiempirical method PM7 and the MOZYME procedure. The calculations were performed in water described with the Conductor-like Screening Model (COSMO) [8], using values of 78.39 and 1.3 Å for the dielectric constant and the effective radius, respectively. From all geometries obtained for a given site, the lowest energy structure was selected.

**Step 3**: For each selected configuration, a partial optimization was performed, with the dsDNA geometry frozen and only PNT relaxed, using the DFT method M062X-GD3/6-31G(d,p) in water (PCM).


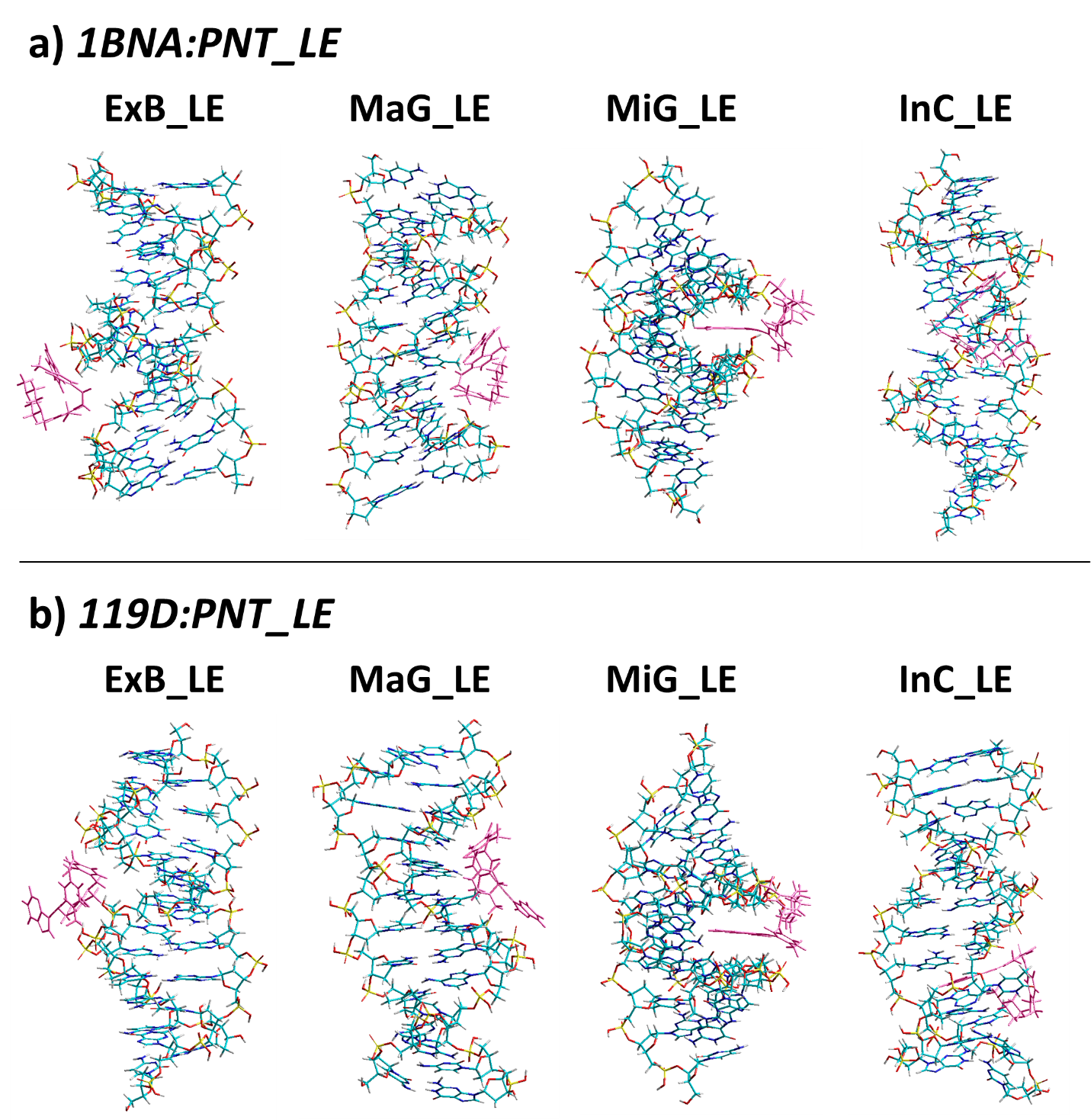


**Figure S3.** Structures of the dsDNA:PNT_LE complexes obtained after the full optimization with the PM7/MOZYME method in water (COSMO). The dsDNA atom colors are: carbon – cyan, nitrogen – dark blue, phosphorus– yellow, oxygen – red, and hydrogen –grey ; the PNT molecule is colored pink.


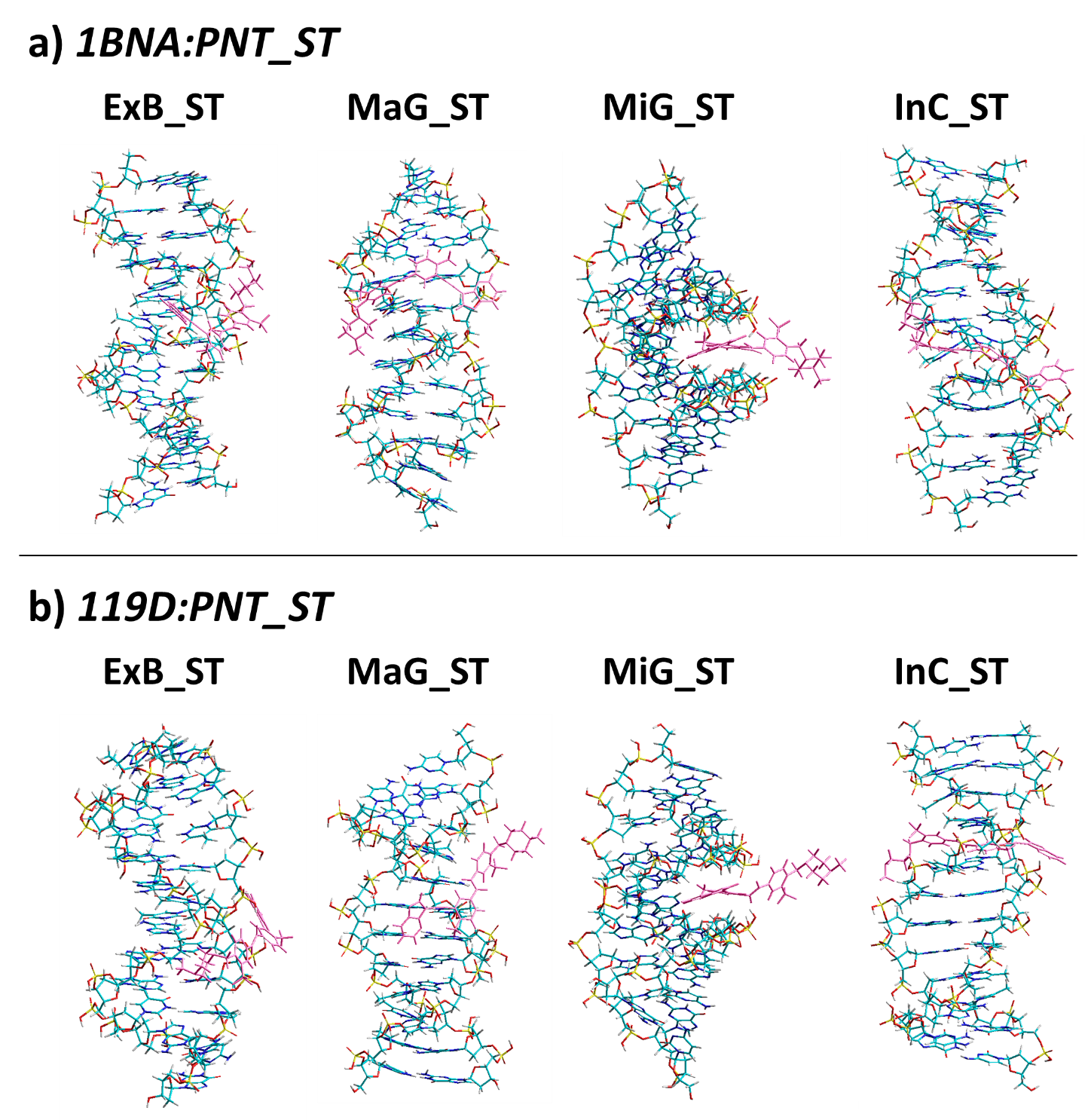


**Figure S4.** Structures of the dsDNA:PNT_ST complexes obtained after full optimization with the PM7 MOZYME method in water (COSMO). The colors are the same as in Figure S2.


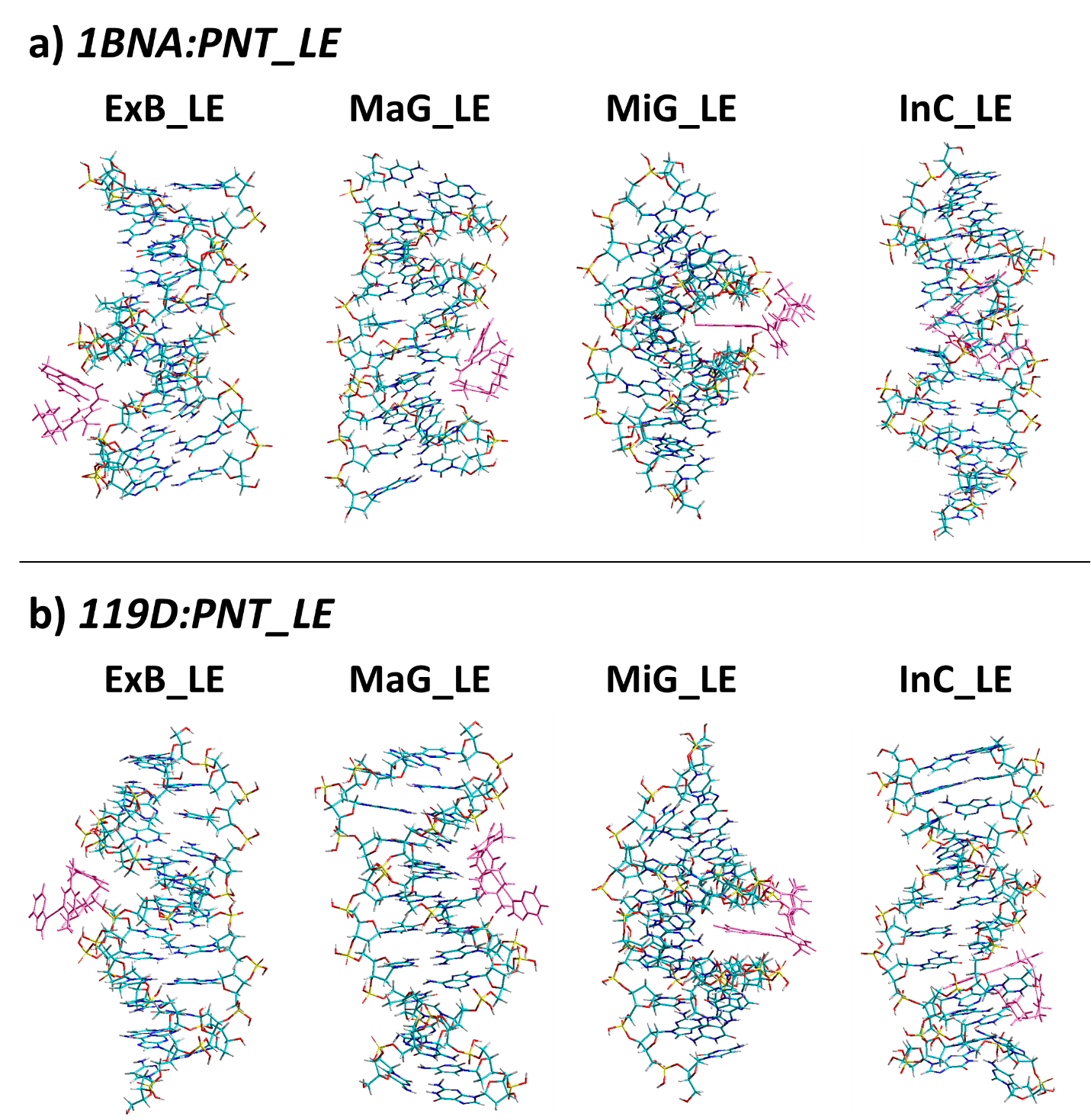


**Figure S5.** Structures of the dsDNA:PNT_LE complexes obtained after partial optimization (dsDNA frozen) with the M062X-GD3/6-31G(d,p) method in water (PCM). The colors are the same as in Figure S2.


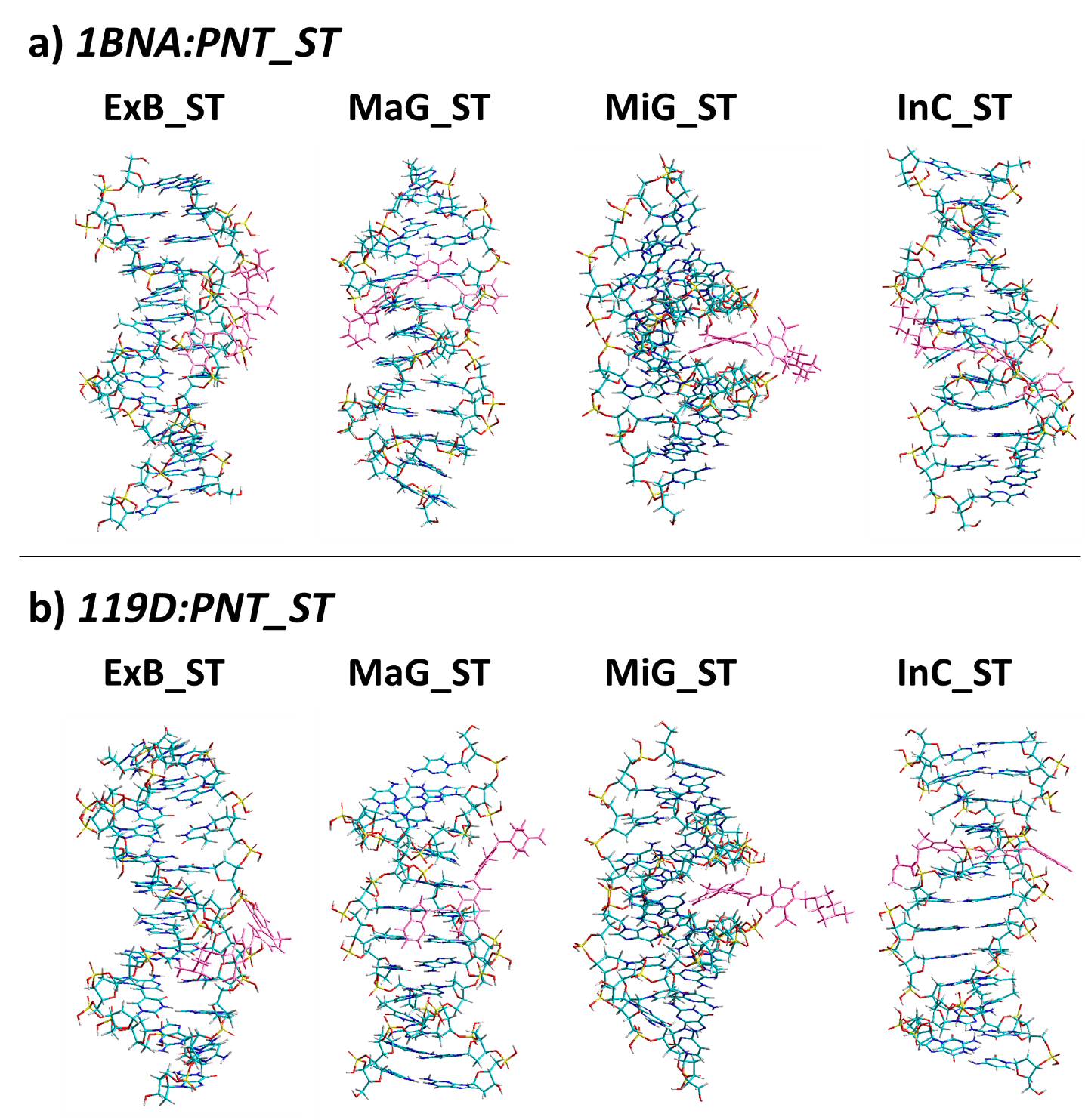


**Figure S6.** Structures of the dsDNA:PNT_ST complexes obtained after partial optimization (dsDNA frozen) with the M062X-GD3/6-31G(d,p) method in water (PCM). The colors are the same as in Figure S2.

**Table S1.** Heats of formation H_f_ were obtained from full optimization with the semiempirical PM7 method in water (COSMO) and total energies E were obtained from partial optimization (dsDNA frozen) with the DFT M062X-GD/6-31G(d,p) method in water (PCM) for the isolated components PNT_LE, 1BNA and 119D and the dsDNA:PNT complexes. All values are in kcal mol^-1^.

|  | **H_f_ (PM7)** | **E (DFT)** | **H_f_ (PM7)** | **E (DFT)** |
| --- | --- | --- | --- | --- |
| **Isolated molecules** | | | | |
| **PNT_LE** | -42.92939 | -1144511.82670 |  |  |
| **dsDNA 1BNA** | -7491.13421 | -20136581.62691 |  |  |
| **dsDNA 119D** | -7473.68184 | -20116411.76863 |  |  |
| **Complexes** | **1BNA:PNT** | | **119D:PNT** | |
| **ExB_LE** | -7567.32705 | -21281118.60716 | -7548.78603 | -21260966.73098 |
| **MaG_LE** | -7604.10579 | -21281150.05862 | -7574.29449 | -21260971.08634 |
| **MiG_LE** | -7588.13469 | -21281143.29162 | -7568.40930 | -21260975.64557 |
| **InC_LE** | -7584.24604 | -21281129.61876 | -7542.69636 | -21260923.79861 |
| **ExB_ST** | -7579.24565 | -21281134.50505 | -7564.33963 | -21260987.30852 |
| **MaG_ST** | -7611.03574 | -21281143.45346 | -7596.78585 | -21260989.21534 |
| **MiG_ST** | -7599.75911 | -21281141.50347 | -7571.54813 | -21260976.18724 |
| **InC_ST** | -7585.76727 | -21281099.95746 | -7574.11834 | -21260983.31599 |

**References**

1. HyperChem(TM) Professional, version 8.0.10. 1115 NW 4th Street, Gainesville, Florida 32601, USA: Hypercube, Inc; 2011.
2. Stewart, J. J. P. MOPAC2016, Stewart Computational Chemistry, 2016, ver. 21.186L, <http://OpenMOPAC.net>.
3. Allouche, R. Gabedit 2.5.1, Gabedit - A Graphical User Interface for Computational Chemistry Softwares. J. Comput. Chem. 2011, 32, 174-182.
4. Frisch, M. J.; Trucks, G. W.; Schlegel, H. B.; Scuseria, G. E.; Robb, M. A.; Cheeseman, J. R.; Scalmani, G.; Barone, V.; Petersson, G. A.; Nakatsuji, H.; Li, X.; Caricato, M.; Marenich, A. V.; Bloino, J.; Janesko, B. G.; Gomperts, R.; Mennucci, B.; Hratchian, H. P.; Ortiz, J. V.; Izmaylov, A. F.; Sonnenberg, J. L.; Williams-Young, D.; Ding, F.; Lipparini, F.; Egidi, F.; Goings, J.; Peng, B.; Petrone, A.; Henderson, T.; Ranasinghe, D.; Zakrzewski, V. G.; Gao, J.; Rega, N.; Zheng, G.; Liang, W.; Hada, M.; Ehara, M.; Toyota, K.; Fukuda, R.; Hasegawa, J.; Ishida, M.; Nakajima, T.; Honda, Y.; Kitao, O.; Nakai, H.; Vreven, T.; Throssell, K.; Montgomery, J. A., Jr.; Peralta, J. E.; Ogliaro, F.; Bearpark, M. J.; Heyd, J. J.; Brothers, E. N.; Kudin, K. N.; Staroverov, V. N.; Keith, T. A.; Kobayashi, R.; Normand, J.; Raghavachari, K.; Rendell, A. P.; Burant, J. C.; Iyengar, S. S.; Tomasi, J.; Cossi, M.; Millam, J. M.; Klene, M.; Adamo, C.; Cammi, R.; Ochterski, J. W.; Martin, R. L.; Morokuma, K.; Farkas, O.; Foresman, J. B.; Fox, D. J. Gaussian 16, Revision C.01, Gaussian, Inc., Wallingford CT, 2016.
5. Zhao, Y.; Truhlar, D. G. The M06 Suite of Density Functionals for Main Group Thermochemistry, Thermochemical Kinetics, Noncovalent Interactions, Excited States, and Transition Elements: Two New Functionals and Systematic Testing of four M06-Class Functionals and 12 Other Functionals. Theor. Chem. Acc. 2008, 120, 215-241.
6. Grimme, S. Semiempirical GGA-Type Density Functional Constructed with a Long-Range Dispersion Correction. J. Comput. Chem. 2006, 27, 1787-1799.
7. Tomasi, J.; Mennucci, B.; Cammi, R. Quantum Mechanical Continuum Solvation Models. Chem. Rev. 2005, 105, 2999-3093.
8. Klamt, A.; Schüümann, G. COSMO: a New Approach to Dielectric Screening in Solvents with Explicit Expressions for the Screening Energy and Its Gradient. J. Chem. Soc., Perkin Trans. 2 1993, 799–805.
